# Preliminary Observations from The FILLED Project (FILipino Lived Experiences during COVID-19)

**DOI:** 10.3390/ijerph191912303

**Published:** 2022-09-28

**Authors:** Melanie D. Sabado-Liwag, Mayra Zamora, Shenazar Esmundo, Jake Ryann Sumibcay, Patchareeya P. Kwan

**Affiliations:** 1 Department of Public Health, California State University, Los Angeles, CA 90032, USA; 2 Department of Health Sciences, California State University, Northridge, CA 91330, USA

**Keywords:** Filipino American, FilAm, COVID-19, health disparities, Asian American, barriers, mental health, culture, healthcare workers, essential workers

## Abstract

Health outcomes for Asian American subgroups are often aggregated, masking unique experiences and disparities exacerbated by the COVID-19 pandemic, specifically among Filipino Americans (FilAms). The FILLED (Filipino Lived Experiences during COVID-19) Project launched a cross-sectional online survey between April-August 2021 among FilAm adults in Southern California to document community issues and outcomes during the pandemic. Among 223 participants, 47.5% were immigrants, 50.9% identified as essential workers, and 40.6% had a pre-existing health condition before the pandemic. Despite high rates of health insurance (93.3%), 24.4% of the sample did not have a regular health care provider. During the pandemic, 32.7% needed mental health help but did not get it and 44.2% did not know where to get such services. Most respondents felt that the COVID-19 vaccination was a personal responsibility to others (76.9%) and the majority had received at least one dose of a COVID-19 vaccine (82.4%). Regarding COVID-19 impact, participants reported moderate-severe changes in their daily routines (73.5%), access to extended social support (38.9%), housing issues (15.4%), and access to medical care (11.6%). To our knowledge, this study is the first community-driven effort highlighting FilAm community experiences in Southern California, where the highest proportion of FilAms in the United States reside, specifically after the COVID-19 vaccine was made widely available. The observational findings may help community leaders, policy makers, and public health researchers in the design, development, and implementation of post-pandemic intervention strategies used by community-partnered projects that address FilAm and sub-Asian group health disparities at grassroots to societal levels.

## 1. Introduction

Despite being one of the largest and most-rapidly expanding ethnic groups, Filipinx/a/o Americans’ (FilAms) health needs are poorly understood [1,2]. FilAms represent the third-largest ethnic minority group and the third-largest Asian American group in the United States (US). They comprise approximately 4.2 million people, yet are dismissed through Asian American aggregate practices in health research and policy [3,4]. Of the 9.8 million people who reside in Los Angeles County, Asian Americans and Pacific Islanders (AAPIs) make up approximately 16% of the population, with approximately 506,000 Filipinos—64% of whom are foreign-born and 19% identify as multiracial [3,4,5]. Often aggregated with other Asian American groups, the social and economic diversity among Asian subethnic groups is masked and overlooked compared to other racial and ethnic groups [6]. Systemically, the pervasive “model minority myth” harms sub-ethnic Asian groups as it universally stereotypes Asians as healthy and smart, erasing the diverse and unique sociopolitical, migration experiences, and/or cultural histories distinguishing Asian Americans across generations [1,6]. Such biases contributes to the lack of in-language and/or culturally appropriate health services and prevention material for immigrant Asian Americans whose primary language is not English; this exclusionary action skews surveillance reports with an abundance of not only English proficient individuals, but with higher income or education attainment, and more likely to utilize services or participate in research.

The COVID-19 pandemic thus revealed and exacerbated existing disparities among ethnic minority and marginalized groups, especially with the allocation of resources, funding, culturally appropriate outreach, and community utilization of preventative services (e.g., screening, vaccinations, etc.) [7]. Specifically, individuals with pre-existing health conditions were at increased risk of COVID-19 infection, hospitalization, intensive care, and death [8,9]. Compared to non-Hispanic Whites, FilAms are at a particularly greater risk of COVID-19-related morbidity and mortality due to disproportionate prevalence of fair to poor health and pre-existing health conditions, such as cancer, obesity, high blood pressure, diabetes, and asthma [10,11]. Furthermore, the adoption of Westernized behaviors and low follow-up appointments after diagnosis have contributed to the growing risk and prevalence of chronic health conditions in the FilAm community [12,13]. For example, FilAm knowledge, attitudes, and behaviors with respect to cancer and cancer prevention are scarcely addressed [14], which ironically is the leading cause of death among FilAms in Los Angeles County where the highest proportion of FilAms reside [15,16]. Underutilization of up-to-date cancer screenings and delays in treatment services are prevalent, which suggests an existing community norm of hesitancy for help-seeking services precluding the consistent use of COVID-19-related resources and services [17,18,19].

Health disparities among FilAm frontline workers worsened during the pandemic [2,20]. In the healthcare industry, FilAms are 4% of the US nursing workforce, yet they accounted for 32% of COVID-19-related deaths by early 2021 [2]. Disparities among FilAm nurses are compounded by pre-existing health conditions and multi-generational living within FilAm communities [3,4,21,22,23]. Additionally, the September 2020 “Sins of Omissions” report found that 83 Filipino registered nurses (26.4%) of 314 registered nurses died of COVID-19 and related complications [24]. Such disparate outcomes in the FilAm workforce are not incidental. Exploitative US immigration policies, generations of Western influence, and systemic traumas forge the conditions in which FilAms represent the most at-risk group of frontline workers during the pandemic [25]. FilAms comprise not only of health care workers but of the foreign-born elderly and other unsung essential workers who unwaveringly adjusted to the complex working environments during the pandemic (e.g., grocery markets, public transportation, caregiving, hospitality, etc.) [26]. As policies and mitigation efforts against COVID-19 evolve, frontline and essential workers carry the brunt of these dynamic working conditions while remaining at risk for excessive psychosocial distress related to uncertain socio-economic hardships (e.g., layoffs, partial or extended hours) [27,28,29]. Furthermore, poor mental health is exacerbated with “stay at home” orders due to the stigma to seek services and/or treatment, as well as addressing or disclosing problems with non-family members often to save face [30,31]. Other intersecting and cumulative stress-inducing situations are often known but less likely discussed among FilAm community households, such as living in multi-generational homes and/or living in close conditions in fear of deportation among undocumented members, in-home violence (e.g., verbal, physical), and taking care of family members (e.g., caregiving, remittance) manifested from cultural values such as utang ng loob (reciprocated debt based on generational gratitude) [32,33,34].

The COVID-19 pandemic brought forward multi-level intersectionalities related to FilAm health disparities. Studies usually report FilAms to have better health outcomes due to their high English proficiency and high educational attainment [35,36]. However, these specific health indicators dismiss and undervalue other determinants that place FilAms high at risk for COVID-19 morbidity and mortality. In addition, the persistent aggregation of Asian and Pacific Islander sub-ethnic groups in data practices ignores the differences in health risks, social variables, and historical conditions across the different groups [1,2,20,37]. Disaggregating data is a pivotal civil rights matter that informs advocates and community members to vouch for policies and budgets to provide more culturally appropriate services for vulnerable and often marginalized AAPI groups [38].

The purpose of this community-led study was to focus on FilAm community experiences during the pandemic to better inform culturally relevant resources and services provided by community organizations that serve and represent them. To this end, we explored the following aims:

1. Capture FilAm specific experiences and behaviors that may be different from aggregate information often reported and concentrated in epidemiologic literature.
2. Identify situational, environmental, and psychosocial factors that contribute to health outcomes and susceptibilities, such as barriers to resources, disruption or limited access to medical care, and other behavioral factors affected by the coronavirus pandemic. Resources include access (and utilization) of medical resources for pre-existing conditions and safe social distanced spaces, financial assistance, and social capital. More relevant to our investigation is observing the access and behaviors directly related to COVID-19, such as frontline or essential work for risk exposure, attitudes towards testing, and thoughts about vaccination with limited information.

## 2. Materials and Methods

This observational, cross-sectional study was initiated by scholars and leaders in the FilAm community to better understand and document behaviors and experiences impacted by the COVID-19 pandemic, one-year post-initiation. This descriptive design was intended to inform development strategies appropriate for community-led interventions and resources. Data was collected from April 2021–August 2021. California State University, Los Angeles Institutional Review Board (IRB) provided expedited approval. Due to COVID-19 risk and various policies set out by the State of California and academic institutions (specifically, research precautions and IRB restrictions), all research activities (e.g., recruitment, consent, and data collection) were mandated to be conducted through non-contact methods. In a collaborative effort, community experts composed of FilAm scholars, stakeholders, and leaders provided feedback regarding relevant questions and constructs. The study was named the FILLED project—FILipino Lived Experiences During COVID-19—to reflect both FilAm-led research efforts and FilAm voices represented through the data, while ‘filling’ the lack of FilAm-data specific information at the height of the pandemic.

**Figure 1:**
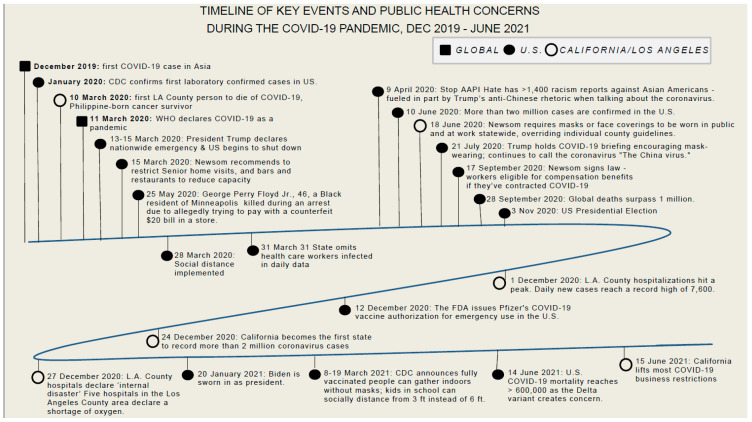
Timeline of key events of Public health Concerns during the COVID-19 pandemic, December 2019–June 2021.

The sample size was determined by nQuery Advisor (version 6) in order to obtain an 80% power to detect any significant effect of individual or community factors of interest at the 0.05 level. Recruitment and data collection approaches were discussed with stakeholders and implemented with their support. Many of the traditional strategies for outreach were limited to remote interactions since community-based organizations (CBOs) were also maintaining contact with their constituents through this method. Moreover, due to pandemic-mandated limitations on traditional public health outreach (e.g., in-person ethnic-related events, festivals, church/religious events, etc.), the study utilized convenience and snowball sampling, a non-probability sampling technique, to identify participants. Potential participants found out about the study through social network referrals (e.g., word-of-mouth, listservs), social media (e.g., Instagram posts and stories), and the FILLED project website which temporarily served as a clearing house for webinars, events, and resources promoted for and by FilAms. Filipino-based community organizations and members throughout the Greater Los Angeles area helped to spread the word through their listservs and social media reposts of flier information. This was imperative to the recruitment and data processes given the complex nature of the pandemic timeframe (Figure 1) [39,40,41,42,43]. This allowed for individuals and networks outside the normal reach of established CBOs to be extended. For example, social network algorithms through Instagram and Twitter helped to link like-minded individuals in the community who wanted to contribute to the work for the community during the pandemic.

To ensure eligibility, potential participants were led to an interest form on the FILLED project website. Participants who identified as 55 years old or older were given the option of completing the survey online or through a mailed paper version of the survey if eligible. Participants were deemed eligible for the online survey if they identified as Filipinx/a/o or Filipinx/a/o American, resided in the Greater Los Angeles area, and were at least 18 years of age. After screening, eligible participants were contacted by the research team with the email address provided in the interest form. A unique online link was sent to each eligible participant, which first ascertained their online informed consent prior to beginning the online survey if they agreed to continue. Data managed through online application systems were password-protected and accessed only by the research team.

The academic research team developed the initial survey’s infrastructure, which was based on a health conceptual model that theorizes that COVID-19 behaviors and health impact is influenced by intersecting factors (internal, environmental, cultural, political) [44]. An interdisciplinary ad hoc group of FilAm health practitioners, researchers, and community leaders were also consulted to review and modify questions for currency and cultural appropriateness given the evolving landscape of the pandemic. Furthermore, community stakeholders and content experts provided feedback during this design process, specifically FilAm elders who agreed that an in-language (Tagalog-translated) version of the survey could be waived at the time and English-only forms would suffice since iterative processes for translation were limited and timing to assess the community was a priority. To offset Tagalog-translated questions, the research processes added the following items to improve outreach and retention: (1) the consent and survey encouraged participants to answer open-ended questions in language if they desired to do so, (2) a survey question asked if they were being assisted, (3) we provided contact information of the research team if they had inquiries when filling out the consent and/or survey, and (4) we distributed a COVID-19 resource list curated by the community and research team to refer direct assistance beyond the study. Along with each outreach email (attachment) and distribution of the study consent (link), we distributed the COVID-19 resource and referral list that included health or social services specifically for AAPIs in the area. Participants were compensated for completing the 30–60 min online questionnaire with over 125 questions. Responses to survey questions about the participant’s background and impacts of COVID-19 (social, physical, and health outcomes) were also obtained. Whether personal protective equipment (PPE) was provided by their employer was also asked, which included masks, face shields, gloves, gowns, sanitizer, and physical distancing measures.

Survey constructs included questions and responses from national surveillance surveys (e.g., Behavioral Risk Factor Surveillance System (BRFSS), National Survey on Drug Use and Health (NSDUH)) and adapted COVID-19-related constructs from the PhenXToolkit promoted by the National Institutes of Health (a web-based catalog of recommended measures). For example, the Kessler Screening Scale for Psychological Distress (K6; *α* = 0.90) is used in BRFSS, an annual general population survey conducted by the CDC. The 6-item scale [45] exhibits appropriate diagnostic sensitivity and specificity across ethnicities [46]. Questions included ‘During the past 30 days, about how often did you feel hopeless?’ and participant responses ranged from ‘All of the time’ (4) to ‘None of the time’ (0). Symptoms of severe psychological distress was defined as a sum score of 13 or higher. [45]

Univariate (i.e., mean, frequencies) statistics are reported for variables measuring sociodemographic characteristics (e.g., age, gender, employment), health status, health care access, and clinical experiences related to COVID-19. As of note, responses to questions regarding health information, as well as attitudes and beliefs about COVID-19 testing and vaccination, were not mutually exclusive (i.e., select all that apply). Given our sample size (*n* = 223), a Shapiro–Wilk test was performed to assess the normality of continuous variables (i.e., age). The distribution of age significantly departed from normality (*W* = 0.83, *p* <0.001). McNemar’s test was used to compare COVID-19 paired responses for pre- and during pandemic. For multivariate regression analyses, models were adjusted for age, gender, education, immigration status, multigenerational households, and job as a frontline or essential worker. Alpha was set at 0.05 and all analyses were performed using SAS version 9.4 [47].

## 3. Results

### 3.1. Sociodemographics

Table 1 describes the characteristics of the sample. The mean age of respondents was 34.2 years (±15.2 SD), age range of 18–75 with about 10% of retirement age and most self-identified as female (68.8%). One-quarter had completed some college and 64.6% had a four-year degree or higher. Seventy percent also reported an affiliation with Christian Catholicism, while 68.3% self-identified as religious and 90.0% identified as spiritual. Approximately less than half (48.0%) of the respondents were born outside of the United States and migrated at the age of 12 or younger (61.3%). At home, 46.6% of respondents spoke English and 51.6% spoke Tagalog or another Filipino dialect. Multi-generational living was prevalent among 36.4% of respondents; 27.2% reported living in close quarters with others in the household. Thirty-three percent were unemployed at the time of the survey, and 36.6% reported student status. Among the employed (66.4%), 57.8% were employed full-time and 18.1% had more than one full-time or part-time job. Only 16.5% were employed with a FilAm-business in the past 12 months from the time of the survey. Among all respondents, about half (50.9%) self-identified as frontline or essential workers. The healthcare industry was the highest-reported employment sector among respondents (31.4%), most of whom (56.4%) worked in a hospital or clinic setting.

### 3.2. General Health and Health Care Access

About 40% reported that they were diagnosed with a pre-existing chronic health condition before the pandemic, while 9.8% reported a new diagnosis during the pandemic. Nearly all respondents (93.3%) had some type of health care coverage and 75.6% had access to a regular health care provider (HCP). Respondents reported poor physical health (62.3%), poor mental health (82.2%), and that poor physical or mental health kept them from doing their usual activities (72.9%) at least one day in the past 30 days. Based on the Kessler-6 psychological scale, an overwhelming majority experienced moderate (50.7%) and severe (22.8%) psychological distress in the past 30 days. More than one-third (32.7%) reported needing but not getting mental health care during the pandemic and 44.2% did not know where to get mental health services.

### 3.3. COVID-19 Impact and Clinical Experiences

Moderate-to-severe changes in several aspects of their lives were reported by respondents as a result of the pandemic (Table 2). These included changes to their daily routines (73.5%), access to extended social support (38.9%), housing issues (15.4%), family income and employment (13.4%), and access to medical care (11.6%). The COVID-19 vaccine was reportedly not offered by employers among 47.7% of respondents. At the time of the survey, less than half (48.9%) reported at least one PPE was provided by their employer. COVID-19 protective measures were fully (45.3%), partially (20.7%), or not at all (1.3%) enforced at respondents’ work sites; some individuals reported not being sure due to their change to remote work (32.7%).

### 3.4. COVID-19 Attitudes and Beliefs

When asked about their most trusted sources for health information, 91.5% of respondents trusted their personal networks (i.e., family, friends, co-workers), 83.9% trusted medical professionals, 65.0% trusted the Internet, and 57.9% trusted health organizations. Respondents self-reported lower perceived threat of COVID-19 at the time of the survey than at the beginning of the pandemic; at the time of the survey, 69.1% reported they were currently concerned about loved ones getting sick with COVID-19, compared to 76.2% at the beginning of the pandemic (*p* = 0.02); 47.5% were concerned about getting sick themselves (vs. 67.7% at the beginning, *p* < 0.0001); and 37.7% avoided people to prevent from getting sick (vs. 51.6% at the beginning, *p* < 0.0001). Only 24.7% said that thinking about COVID-19 made them feel threatened (vs. 35.4% at the beginning, *p* = 0.002), and 24.3% did not believe COVID-19 was a threat to them or their family at the time of the survey (vs. 18.0% at the beginning, *p* = 0.10). In addition, vaccinated individuals were more likely to be concerned about getting sick themselves (aOR = 2.75, 95%CI = 1.20–6.35, *p* = 0.02) and actively avoided others to prevent from getting sick (aOR = 3.26, 95% CI = 1.42–7.49, *p* = 0.005).

### 3.5. Screening: COVID-19 Testing

At the time of the survey, 76.2% tested for COVID-19 at least once and 14.0% tested positive during the pandemic. Among respondents who had previously tested for COVID-19, 36.3% tested as a precautionary measure after attending a social gathering, 34.1% were required or recommended by their employer to get tested, and 33.2% had been exposed to someone who tested positive for COVID-19. Respondents who had tested for COVID-19 reported initial hesitations, including not feeling the need to test (33.6%), fear of a positive test result (14.8%), and not being able to schedule an appointment soon enough (14.8%). Among respondents who had never tested for COVID-19 (18.1%) at the time of the survey, 10.3% indicated that they had been feeling well and 9.9% did not feel the need to since they had not been in contact with a COVID-19-positive individual.

### 3.6. COVID-19 Vaccinations

The majority of respondents (74.1%) reported that they regularly followed general vaccination guidelines before the pandemic, and another 21.8% sometimes or often followed guidelines. Most participants (82.4%) received at least one dose of the COVID-19 vaccine, with 38.4% receiving their vaccine(s) at a community clinic or hospital and 35.1% from a Public Point of Distribution (POD) site organized by federal, state and/or county entities. Among vaccinated respondents, initial hesitations towards the vaccine included concerns about side effects (51.6%), concerns about the vaccine being “too new” (21.5%), and a general aversion to shots (8.1%). Among the 17.6% unvaccinated individuals at the time of data collection, hesitancies included concerns about side effects (12.1%), concerns about the vaccine being “too new” (9.4%), and concerns over the role of politics in the vaccine’s development (4.5%). When scheduling their vaccine appointment, 80.9% of vaccinated respondents scheduled their own appointment, while 21.4% had a family or friend help them schedule their vaccination appointment and 16.3% assisted others when scheduling their appointment.

Those with a pre-existing chronic health condition diagnosis before the pandemic were more likely to have received a COVID-19 vaccination than those who did not have a chronic health diagnosis before the pandemic (aOR = 3.11, 95%CI = 1.25–7.75; *p* = 0.01). Among those who did not have a pre-existing condition (59.4%), unvaccinated individuals were less likely to report concern being around people and catching COVID-19 compared to those vaccinated (aOR = 0.18, p6%CI = 0.06–0.60, *p* = 0.005).

Overall, 76.9% of respondents perceived vaccination against COVID-19 as a personal responsibility to protect the health of others. When asked about recommendations for decreasing vaccine hesitancy in the FilAm community, respondents advocated for increased messaging to combat COVID-19 misinformation (74.4%). They also advocated for more culturally tailored information about the impact of COVID-19 on the community (65.0%), encouragement from HCPs (63.7%), no cost to vaccinations (59.2%), more in-language material about COVID-19 (58.3%), and more discussions within families about COVID-19 (58.3%).

## 4. Discussion

The FILLED project results provide preliminary insights into FilAm issues one year into the pandemic. Similarly and subsequently, the study occurred after many FilAm health and essential workers’ lives were lost, the healthcare system experienced some reprieve with the US Food and Drug Association (FDA) emergency approvals of Pfizer and Moderna COVID-19 vaccinations, and the belief that normalcy would soon return with herd immunity. The COVID-19 pandemic provided an opportunity for communities to leverage dismissed existing health disparities among minority groups to garner allyship, warranted attention, and needed resources back to the community. There are a number of insights to note from this observational study, both from its results and from the research process in this community-academic effort. To our knowledge, this is the first community-based cross-sectional survey created for and by FilAms when the COVID-19 vaccine became widely available to all Americans 15 years old and older.

### 4.1. Compounding Risk Factors and Layering Protective Measures

FilAm frontline workers often live and function in multi-generational households. With the ‘Shelter at Home’ order, caution and thoughtful planning were implemented to protect family members at higher risk of contracting COVID-19 while dealing with other possible health issues. Although not generalizable, our study corroborates the findings from the Pew Research Center, where over a third of FilAms live in multigenerational households, especially during the pandemic [4]. Adding another layer of complication to COVID-19′s impact is the determinant of a pre-existing chronic health condition diagnosed prior to the pandemic, which claimed roughly 40.6% of our sample. Prior to the pandemic, low-income and communities of color were highly susceptible to adverse health outcomes, which are highly contributed by lack of resources, poor built-in and/or living environments, and cultural determinants. The imminent risk of getting COVID-19 while immunocompromised was compounded with existing factors related to increased morbidity and mortality among minority, underserved, and marginalized communities, such as those from the Asian American community. In addition, 10% of our sample was newly diagnosed with a chronic condition during the pandemic; whether the condition was unrelated or related to long-COVID-19 was not assessed. Our results are evident of compounding and heightened risk, especially where half reported being an essential worker (with 31.4% in the healthcare industry) and 17.6% were unvaccinated at the time of the study despite the vaccine being available to them. Low PPE access and weak enforcement of COVID-19 protective measures by employers were raised to be an issue one-year into the pandemic, especially in California where health policies and mandates often set precedents. Our study findings are in line with a report that revealed that Filipino frontline workers at residential care facilities for the elderly experienced a lack of health and safety protections and that these facilities were “hot spots” for the spread of COVID-19 between workers and patients [22]. Furthermore, adopting multi-layer protective measures (e.g., COVID-19 vaccinations, masking, and physical distancing) for high-traffic spaces to protect at-risk individuals such as FilAms with preexisting conditions working in healthcare settings and living in multigenerational household were imperative for slowing down COVID-19 spread and increasing prevention [48,49].

Moreover, a strong majority of study participants were vaccinated against COVID-19 and believed vaccination was a personal responsibility, thus affirming the collectivism present within FilAm communities. Although this sample was highly educated and expressed favorable views towards vaccination, over one-third of our sample reported needing help or having helped someone schedule a vaccination appointment. Opportunities, thus, abound for improving resources and outreach efforts to make COVID-19 vaccines more easily accessible, and such opportunities can amplify FilAm voices within their own households and communities.

To achieve greater equity and ensure preventative measures are accepted normatively, like vaccinations among other at-risk ethnic and community-oriented communities, our study participants advocated for a number of ways public health efforts can be improved. These included recommendations for culturally tailored information about COVID-19′s impact, in-language materials about COVID-19, and family discussions about the vaccine. With more than half of our sample speaking another language other than English at home, such recommendations provide valuable insight into specific action steps policy makers, public health advocates, and community health workers can all take to decrease vaccine hesitancy among at-risk communities.

Furthermore, an overwhelming proportion of participants shared that they perceived doctors or medical professionals as trusted sources of health information, and over two-thirds recommended that health care providers should encourage patients to get vaccinated as a means of further increasing vaccine uptake in the FilAm community. Given the level of misinformation during the pandemic, which was eventually coined as an ‘infodemic’ (defined by the World Health Organization as the inundation of COVID-19 information that included false information that could mislead communities) [50], there is an opportunity for healthcare providers to be utilized as better resources and vessels for positive COVID-19 behaviors (e.g., testing, vaccinations) and other health-related advice (e.g., chronic disease screenings, follow-up or treatment for poor mental health). Ironically, it should be assumed that if one in four FilAms are employed in the healthcare field, the FilAm community should be doing better in obtaining proper healthcare advice. Despite being a highly vaccinated sample, the issues surrounding vaccine hesitancy among FilAm healthcare workers have yet to be explored, especially given that 85.7% of the sample reported Christianity (e.g., Catholicism, other) as their affiliated religion and 68.3% defined themselves as religious. Any correlations may be anecdotal given the polarizing political landscape during the pandemic.

### 4.2. Social Support and Mental Health

The highest area of COVID-19 impact for the FilAm community was access to extended social support where many reported moderate-to-severe changes. For this culturally collectivistic community, a disruption in social support may have affected many individuals’ coping strategies during the pandemic. The ‘Stay at Home’ order may have physically saved lives and/or reduced the level of burden on the healthcare system, but it created an evitable toll on communities who rely on community networks and feeling connected for psychological wellbeing. Increasing Zoom, video-conferencing, and technical support was an area that most CBOs and individuals jumped into action to address, especially amongst older members of the community who were at-risk for social isolation. The reliance on technology to stay connected during the pandemic was a major unavoidable shift that many were forced to trust and adapt to.

Further underscoring the pandemic’s impact on social supports and the importance of meaningful dialogue on mental health among FilAms is the finding that 32.7% of our sample reported needing mental health services during the pandemic and not receiving any. Although FilAm mental health is obtaining more traction in the literature, the community still has a long way to go to combat the level of social and personal stigma as well as systemic discrimination when speaking and/or seeking health services in general. There are deep-rooted systemic factors that contribute to FilAms’ health disparities and negative mental health outcomes, such as intergenerational, complex experiences of historical imperialism policies, colonialism, racism, and colorism [51,52,53]. Given the intersectionalities of structural barriers, comorbid conditions, frontline or essential work, socioeconomic and environmental challenges created by the pandemic, loss of social capital, and rising incidents of anti-Asian hate, poor mental health is a long-term health outcome that needs to be better assessed and addressed swiftly with cultural humility in order to prevent or delay burn-out and further psychological distress.

### 4.3. Community Assets and Strengths for Health Equity Research

There are also relevant takeaways from community-engaged research processes during the pandemic. Community-based participatory research, community-partnered participatory research, and/or any community-engaged research using stakeholders throughout the research process during the pandemic was challenging for all parties involved. It is important to acknowledge the extensive strain placed upon CBOs during the pandemic as they rerouted resources and made specific adjustments to protect both themselves and their at-risk communities. This meant pausing regular pre-pandemic services that often relied on in-person and social interactions (e.g., naturalization facilitation, art/cultural exhibits). As funding became available for COVID-19-related services and resources, as well as frontrunners for trusted COVID-19 information became apparent, CBOs quickly adjusted to using Zoom or online platforms for social gatherings or get-togethers for remote interactions since the onset of ‘Stay at Home’ orders in mid-March 2020.

The idea for community assessment among community partners was discussed in the summer preceding the 2020 general election. It was intentionally decided to delay recruitment and data collection until after the January 2021 presidential inauguration. This timing decision was intended to offset the heightened anxiety and stress that resonated nationally around socio-political tensions brought on by the general election. Although potential participants’ mental health status was considered, this decision also came with unforeseen challenges. Despite the commendable remote adjustment worldwide, approaching primary research one-year into the pandemic meant the inevitable existence of cumulative pandemic barriers. For example, although people may have ‘wanted to participate’ based on their initial contact and interest, many individuals failed to follow-through to complete the survey. Participants, many of whom were snowball sampled, were respectfully followed-up per the set research protocol. A few months after the end of data collection, a virtual town hall was hosted to share initial results with the community. Anonymous sources stated ‘time’, ‘online fatigue’, and ‘prioritizing offline activities’ as reasons to not complete the survey. In addition, other larger COVID-19 studies were being launched and promoted coincidentally at the same time as our data collection. This may have impacted our recruitment and retention.

During post-project analysis and reflection with our research team and with community stakeholders, we quickly realized the consequence of launching the study when the Pfizer and Moderna COVID-19 vaccines became widely available to all levels of the US community—at-risk, healthy and non-at-risk individuals, alike. This meant that society became more confident to reclaim pre-pandemic activities. Although many researchers would see this as a limitation, our team chose to accept this barrier to attaining a larger sample size as a positive determinant for better health outcomes for the community. To note, development and IRB approval of the study occurred when vaccinations were not fully approved outside the efficacy stage.

### 4.4. Study Limitations

A majority of the sample resided in the Greater Los Angeles area; therefore, our observational reports may not be generalizable to FilAms outside of this geographic area. The assessment was conducted in English-only, which may have discouraged limited-English proficient FilAms from participating if they did not have assistance or support to join the study. Additionally, all study-related activities were performed remotely, which required participants to have updated technology (e.g., smartphone, laptop, and/or computer) with reliable WiFi or Internet to access the online interest form, consent, and/or survey, as well as email for correspondence. Although survey questions and constructs were consulted with FilAm stakeholders, not all questions were culturally adapted or modified, and standard instruments were used. Less was known about COVID-19 survey instruments during the development of this survey. Although the desired sample size of 300 was not achieved which would have helped with further comparative analyses, we are confident that the observational information ascertained among FilAms in California during a critical phase of the pandemic (post-mass vaccination availability) can be utilized to confirm the need for specific community interventions. Despite the small sample size and initial convenient recruitment approach, the current study findings corroborate other studies regarding FilAm health-related issues, while bringing to light COVID-19 behaviors, attitudes, and environment during the pandemic.

### 4.5. Future Directions

Post-pandemic research considerations may include modifying a data infrastructure that collects and disseminates disaggregated Asian-subgroup information around COVID-19-related health outcomes when possible. Aggregated information continues to mask the differences and unique strategies needed for optimal health post-pandemic for each community. Homogenizing is a form of structural discrimination against Asian Americans and each subgroup. Maintaining discriminatory policies and systemic biases that favor more accessible communities who are more likely to participate in research and clinical trials perpetuates the model minority myth that Asians are healthier and do not experience disparities. This extends and further restricts policies related to proper funding allocation and stunts representation of these communities to combat structural health inequities often felt at community and personal levels. While there is an elevated commitment to disaggregating data within the healthcare system, public health, and other sectors, the current study would not have been possible without the community-driven motivation to explore further the limited but notable findings of FilAm health disparities through disaggregated data. This project made deliberate efforts to build upon existing CBO relationships, while establishing ties with new FilAm, Asian, and Pacific Islander advocacy groups and reconnecting with other camaraderies aimed at the same COVID-19 goal: to serve and give voice to the Asian and FilAm communities. With granular data and evidence, stakeholders can identify clear pathways to effectively intervene and shape policies and practices that promote health and health equity [54]. In addition, more focus should continue to understand the impact among FilAm essential and frontline workers, especially a minoritized subgroup often unsung-FilAm-owned businesses and those employed by them. During the pandemic, these FilAm establishments represented resiliency and commitment to continue serving and providing a central place for cultural connectivity, even if limited through food.

## 5. Conclusions

FilAm scholars partnered with seasoned community advocates to produce the FILLED project survey, which was inherently designed to assess and document COVID-19 behaviors and experiences, comprehensively, for real-time community use. At the time of the survey, larger epidemiologic surveys and big data strategies on Asian Americans helped to inform and validate our community-partnered project, but less was known about the community challenges and barriers to data collection. Our survey findings and research processes highlight the importance of community-driven approaches and methodologies in order to document existing and newly emerging issues among Asian subgroups, like FilAms. Through this study, we confirmed the importance of maintaining community-engaged partnerships for health equity research not only to inform the development of assessments, interventions and services, but also to further amplify community assets and resiliency as characteristics for building capacity and awareness among marginalized groups.

## Figures and Tables

**Table 1:** Demographic characteristics of the Filipinx/a/o American sample (n = 223).

	n	%
**Age** (mean (±SD))	34.2 (±15.2)	
18–24	71	33.02
25–34	68	31.63
35–44	31	14.42
45–64	21	11.17
>64	21	9.77
**Gender**		
Female	152	68.78
Male	66	29.86
Other	3	1.36
**Education**		
<High school graduate	23	10.45
Some college/trade school/2-year college grad	55	25.00
4-year college grad or more	142	64.55
**Marriage status**		
Single	127	56.95
Married	70	31.39
Other	26	11.67
**Born in the US**	115	52.04
**Age moved to US**		
<12 years old	65	61.32
**Religion**		
No affiliation	24	10.76
Christian Catholic	156	69.96
Other Christian	35	15.7
Other	6	2.69
**Religious**		
Yes, definitely—maybe yes	151	68.33
Definitely not—maybe no	70	31.57
**Spiritual**		
Yes, definitely—maybe yes	199	90.05
Definitely not—maybe no	22	22.00
**Employed**	148	66.37
**Number of paid jobs current/during pandemic**		
1 Full-time	96	57.83
1 Part-time	22	13.25
2+ Full-time and/or Part-time	30	18.07
Retired or homemaker	18	10.84
**Current student** (full/part time)	87	36.65
**Work for Filipino-owned business (past 12 months)** ^a^	36	16.52
**Frontline or Essential Worker**	106	50.96
**Employment industry**		
Healthcare	69	31.36
Non-Healthcare	151	68.64
**Language spoken at home**		
English	103	46.61
Tagalog or another Filipino dialect	114	51.59
Other	4	1.81
**Living situation during COVID-19**		
Own	54	24.43
Rent	76	34.39
Live with family/parents—before and during COVID-19	82	37.10
Other ^b^	9	4.06
**Living in multi-generational household**	80	36.36
**Close living conditions during COVID-19**	60	27.15
**Diagnosed with a chronic health condition pre-COVID**	87	40.65
**Has health insurance**	208	93.27
**Access to a regular health care provider**	167	75.57
**Access to a regular holistic health care provider/traditional healer**	43	19.46

Note that single response lines are binary (‘yes’/’no’) with count and percent shown for ‘yes’ responses. ^a^ Responses include individuals who stopped working for a FilAm-owned business due to the COVID-19 pandemic mandated shut-downs in Los Angeles County and throughout California in early 2020. ^b^ Responses collapsed into ‘Other’ category for ‘*Living situation’* include ‘Living/staying with parents or other family members only during COVID-19; I have another place of residence in which I rent or own’, ‘living with employer’, and ‘other arrangement.’

**Table 2:** Descriptive observations of COVID-19 impact on employment, screening, vaccination, social, health care access, physical health, and mental health.

	n	%
**WORK-RELATED IMPACT** **Filed for unemployment during COVID-19**	37	20.44
**Ever afraid of losing their job** (if employed during the pandemic)		
Yes, very—moderately stressful	51	33.78
Yes, but not very stressful	20	13.25
No	80	52.98
**PPE offered by employer** (if worked in-person)		
Yes (at least 1 type ^ of PPE)	109	48.88
None (supplied own)	114	51.12
**COVID-19 protective measures enforced by employer**		
Fully enforced	68	45.33
Partially enforced	31	20.67
Not enforced at all	2	1.33
Not sure, working remotely	49	32.67
**Pandemic changed outlook of current job/profession**		
No change	62	32.12
Mild positive change	57	29.53
Mild negative change	42	21.76
Moderate change	22	11.40
Severe change	10	5.18
**SCREENING: TESTING FOR COVID-19** **Self-reported frequency of COVID-19 testing**		
Never tested	39	17.49
5 times or fewer	123	55.16
More than 5 times	47	21.08
**Tested positive for COVID-19**	31	13.9
**VACCINATION**		
**Followed medical guidelines for vaccinations before COVID-19 pandemic**		
Always	160	74.07
Sometimes	35	16.20
Often	12	5.56
Avoid vaccinations	9	4.17
**Attitudes toward COVID-19 vaccination**		
Personal choice	50	23.15
Personal responsibility to others	166	76.85
**COVID-19 vaccine offered by employer**		
Yes	88	41.12
No	102	47.66
Don’t know	24	11.21
**Vaccinated against COVID-19** (if vaccinated)		
Yes—two doses	140	64.81
Yes—one dose	38	17.59
No—appointment pending	10	4.63
No—appointment not yet scheduled	16	7.41
No—no plans to get vaccinated	12	5.56
**COVID-19 vaccination site** (if vaccinated)		
POD (Public Point of Distribution)	65	35.14
Community clinic or hospital	71	38.38
Pharmacy	34	18.38
Not sure	15	8.11
**Top reasons for getting COVID-19 vaccination** (if vaccinated)		
My social responsibility to protect my community	160	89.89
Need to protect my family	157	88.20
Need to protect myself	155	87.08
Other ^a^	119	66.85
**OVERALL COVID-19 IMPACT** **Daily routine**		
No—mild change	21	9.77
Mild change	36	16.74
Moderate change	73	33.95
Severe change	85	39.53
**Family income/employment**		
No change	71	32.87
Mild positive change	55	25.46
Mild negative change	61	28.24
Moderate change	22	10.19
Severe change	7	3.24
**Housing situation**		
No change	141	65.58
Mild change	41	19.07
Moderate-severe change	27	12.56
Severe change	6	2.79
**Access to food**		
No change	140	65.12
Mild change	56	26.05
Moderate change	18	8.37
Severe change	1	0.47
**Access to medical health care**		
No change	94	43.52
Mild change	97	44.91
Moderate change	21	9.72
Severe change	4	1.85
**Access to extended social supports**		
No change	53	24.54
Mild change	79	36.57
Moderate change	72	33.33
Severe change	12	5.56
**Family stress and discord**		
No change	89	41.2
Mild change	95	43.98
Moderate change	29	13.43
Severe change	3	1.39
**Challenges to accessing healthcare**		
Delays in calling my health provider (i.e., long call wait times)	27	12.11
Delays in getting referrals or seeing specialists	26	11.66
Difficulty in initially using telehealth	12	5.38
Issues getting lab tests or screened for health conditions	11	4.93
Other ^b^	12	5.38
**Trusted sources for health information**		
Doctor or medical professional	187	83.86
Internet	145	65.02
Health organization or agency	129	57.85
Family	108	48.43
Printed materials	94	42.15
Friend/Co-worker	96	43.05
Coach, mentor, or community elder	27	12.11
Other ^c^	34	15.24
None of the above	4	1.79
**Needed mental health help but didn’t get it (since 15 March 2020)**	73	32.74
**Do not know where to get mental health services**	96	44.24
**Poor physical health** ^1^	137	62.27
**Poor mental health** ^1^	180	82.19
**Physical/mental health prevent usual activities** ^1^	159	72.94

Note that single response lines are binary (‘yes’/’no’) with count and percent shown for ‘yes’ responses. ^ Types of PPE include N95 filtering face piece respirator or higher masks, surgical/cone-style or cloth masks, face shields, goggles, gloves, gowns, hand washing stations, hand sanitizer, and physical distancing measures (policy reminders and signs). ^a^ Responses collapsed into ‘Other’ category for ‘*Main reasons for getting the COVID-19 vaccination*’ include protecting people in the workplace, highly suggested by their employer, the vaccine was free, felt pressure from family/peers, and others. ^b^ Responses collapsed into ‘Other’ category for ‘*Challenges to healthcare access*’ include delays in prescriptions and/or refills, and medical team or other patients did not follow protective guidelines (if attended in person). ^c^ Responses collapsed into ‘Other’ category for ‘*Trusted source for health information*’ include telephone hotline, traditional healer, and spiritual or religious advisor’ ^1^ Health related quality of life was evaluated as 1 or more days in the past 30 days.

## Data Availability

Not applicable.
